# Live birth outcomes from IVF treatments in younger patients with low AMH

**DOI:** 10.5935/1518-0557.20210006

**Published:** 2021

**Authors:** Maho Miyagi, Keiko Mekaru, Rie Nakamura, Sugiko Oishi, Kozue Akamine, Chiaki Heshiki, Yoichi Aoki

**Affiliations:** 1 Department of Obstetrics and Gynecology, Graduate School of Medicine, University of the Ryukyus. Nishihara, Okinawa, Japan

**Keywords:** anti-Müllerian hormone, *in vitro* fertilization, ovarian reserve, live birth, oocyte retrieval

## Abstract

**Objective:**

Anti-Müllerian hormone (AMH) is used to predict in vitro fertilization outcomes. However, predicting live birth is difficult in younger patients with low AMH. Thus, this study aimed to determine the live birth rates from younger patients with low anti-Müllerian hormone levels.

**Methods:**

A total of 296 infertile patients with AMH measured (younger group, aged 25-38 years; older group, aged 39-42 years) were included in this study. In vitro fertilization outcomes between patients with AMH levels of <1.0ng/mL and ≥1.0ng/mL were compared.

**Results:**

Younger patients with AMH levels <1.0ng/mL (younger low AMH group) exhibited lower number of oocytes retrieved than patients with AMH levels ≥1.0ng/mL (younger normal AMH group). However, there were no significant differences in cumulative pregnancy or cumulative live birth rates between groups. Older patients with AMH levels ≥1.0ng/mL (older normal AMH group) had significantly better outcomes as per mean number of oocytes, cumulative pregnancy rate, and cumulative live birth rate than older patients with AMH levels <1.0ng/mL (older low AMH group). In the younger low AMH group, the frequency of oocyte retrieval was significantly higher in patients who achieved live birth. In addition, the blastocyst transfer rate was significantly higher in individuals with live births versus subjects with non-live births.

**Conclusions:**

AMH is a predictor of live birth among older, but not younger, women. Our report suggests that younger women may become pregnant even with low AMH levels when they obtain blastocysts from frequent oocyte retrievals.

## INTRODUCTION

Anti-Müllerian hormone (AMH) is an accurate predictor of a woman’s ovarian reserve. It has been used to predict the outcomes of assisted reproductive technology (ART) and to determine treatment regimens. AMH is secreted by granulosa cells in women, and its levels in blood reflect the number of secondary follicles, with decreases observed with aging (Tal *et al*., 2015). Although AMH levels also correlate with the number of developing follicles due to ovarian stimulation, predicting pregnancy and live births using AMH levels is difficult (Tal *et al*., 2015; La Marca *et al*., 2010). In younger patients in particular, live births may reportedly be achieved even with low AMH levels (Sefrioui *et al*., 2019). In general, patients with low AMH levels are difficult to treat because the number of oocytes retrieved is low (Baker, 2018; La Marca *et al*., 2007). Some reports indicate that AMH levels are not related to live birth or pregnancy rates (Tal *et al*., 2015; Hansen *et al*., 2016). This study aimed to determine the live birth rate in younger patients with low AMH, and to further identify the factors tied to how live births were achieved in this group.

## MATERIAL AND METHODS

A total of 296 infertile patients aged 25-42 years were included in this study. Their AMH levels were measured at the time of first oocyte retrieval at our department between January 2015 and December 2017. Around 1077 cycles were recorded.

**Figure 1:**
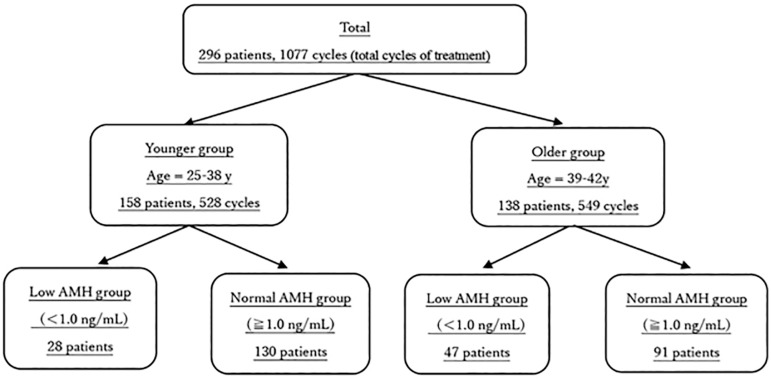
Flowchart of setting the target population.

In vitro fertilization (IVF) outcomes in patients with an AMH level <1.0ng/mL and ≥ 1.0 ng/mL were evaluated retrospectively using our institution’s databases and medical records of ART treatment. Patients were divided into two groups: the younger group (aged 25-38 years) and the older group (aged 39-42 years) (Fig. 1). ART methods included IVF and intracytoplasmic sperm injection, fresh embryo transfer, and thawed embryo transfer. The mean number of oocytes retrieved per oocyte pick-up cycle, the mean frequency of oocyte retrievals per patient, the cumulative pregnancy rate per patient, and the cumulative live birth rate per patient were compared and evaluated across the younger low (AMH <1.0ng/mL), younger normal (AMH ≥1.0ng/mL), older low (AMH <1.0ng/mL), and older normal (AMH ≥1.0ng/mL) AMH groups.

For the ovulation induction method, a short protocol or antagonist protocol was utilized in the ovarian stimulation protocol of patients with normal ovarian function. A mild stimulation protocol, such as with clomiphene citrate and a natural cycle, was prescribed to patients with decreased ovarian function. AMH was examined using an electrochemiluminescence assay.

A *t*-test or Fisher’s exact test was used in statistical analyses, and a *p*-value of < 0.05 was considered statistically significant. Figure 1 illustrates the study design.

## RESULTS

The mean age of the patients was 37.8 years, and the median AMH level was 1.5ng/mL. The pregnancy rate per embryo transfer in all patients was calculated as 28.2 % (153/543 cycles), and the live birth rate per embryo transfer was 19.4 % (106/543 cycles). The median AMH value was 0.61ng/mL in the younger low AMH group (range: 0.00-0.99ng/mL; high: AMH >0.74ng/mL; medium: 0.50-0.74ng/mL; and low: AMH <0.50ng/mL) and 2.67ng/mL in the younger normal AMH group (range: 1.00-23.00 ng/mL; high: AMH >5.00 ng/mL; medium: 5.00-2.00ng/mL; and low: AMH <2.00ng/mL). The median AMH level was 0.43ng/mL in the older low AMH group (range: 0.00-0.99ng/mL; high: AMH >0.75ng/mL; medium: 0.75-0.43ng/mL; and low: AMH <0.43ng/mL ) and 2.22ng/mL in the older normal AMH group (range: 1.00-14.50ng/mL; high: AMH >4ng/mL; medium: 2.00-4.00ng/mL; and low: AMH <2ng/mL).

Table 1 presents the IVF results for the younger low AMH group and the younger normal AMH group. Although the mean number of oocytes retrieved was significantly higher in the younger normal AMH group than in the younger low AMH group, no significant differences in pregnancy or live birth rates per patient were noted. Mean total HMG doses and the average estradiol levels were significantly higher in the younger normal AMH group than in the younger low AMH group. In addition, the younger low AMH group exhibited a significantly higher mean frequency of oocyte retrievals per patient than the younger normal AMH group.

**Table 1:** IVF outcomes in the younger low AMH and younger normal AMH groups.

	Younger Low AMH group * (n=128 cycles)	Younger Normal AMH group ** (n=400 cycles)	p
Age (y), mean ± SD	35.2±0.48	34.8±0.27	N.S.
AMH (ng/mL), mean ± SD	0.49±0.30	4.17±0.17	<0.0001
Total doses of HMG/ FSH (IU) mean ± SD	1523.4±114.1	1982±52.7	0.0003
Estrogen value of before hCG (pg/mL), mean ± SD	518±182.5	1317±98.8	0.0001
Progesterone value of before hCG (ng/mL), mean ± SD	0.7±0.09	0.6±0.05	N.S.
No. of oocytes retrieved, mean ± SD	2.8±1.63	11.5±1.13	0.0001
Frequency of oocyte retrievals per patient, mean ± SD	2.6±0.38	1.3±0.18	0.0001
Cumulative pregnancy rate per patient	50% (14/28)	65.9% (95/144)	N.S.
Cumulative live birth rate per patient	39.3% (11/28)	50% (72/144)	N.S.

* Low AMH: AMH <1 ng/mL.

** Normal AMH group: AMH ≥1 ng/mL.

AMH=anti-Müllerian hormone; IVF=in vitro fertilization; N.S.=not significant.

Table 2 shows the IVF results in the older low AMH group and older normal AMH group. The mean number of oocytes retrieved, pregnancy rate per patient, and live birth rate per patient were higher in the older normal AMH group. Similar to the findings on the younger groups, the mean total HMG dose and the average estradiol level were significantly higher in the older normal AMH group than in the older low AMH group.

**Table 2:** IVF outcomes in the older low group and older normal group.

	Older Low AMH group* (n = 173 cycles)	Older Normal AMH group** (n = 376 cycles)	*p*
Age (y), mean ± SD	40.6 ± 0.16	40.7 ± 0.11	N.S.
AMH (ng/mL), mean ± SD	0.45 ± 0.16	2.99 ± 0.11	<0.0001
Total doses of HMG/ FSH (IU), mean ± SD	1487 ± 113	1816 ± 66.5	0.013
Estrogen value of before hCG (pg/mL), mean ± SD	481 ± 86.1	927 ± 60.4	<0.0001
Progesterone value of before hCG (ng/mL), mean ± SD	0.35 ± 0.04	0.49 ± 0.03	0.0076
No. of oocytes retrieved, mean ± SD	1.96 ± 1.08	6.83 ± 0.83	<0.0001
Frequency of oocyte retrievals per patient, mean ± SD	2.43 ± 0.52	1.98 ± 0.38	N.S.
Cumulative pregnancy rate per patient	14.6 % (7/48)	19.8 % (37/96)	0.0038
Cumulative live birth rate per patient	6.3 % (3/48)	20.8 % (20/96)	0.0292

* Low AMH, AMH <1 ng/mL.

** Normal AMH group, AMH ≥1 ng/mL.

AMH=anti-Müllerian hormone; IVF=in vitro fertilization; N.S.= not significant.

Table 3 shows the comparisons between patients who had live births and the subjects unable to attain live births in the younger low AMH group. In the younger low AMH group, the mean frequency of oocyte retrievals per patient was statistically higher in patients with live births (3.5) than in patients unable to achieve live births (2.1) (*p*=0.035). In addition, the blastocyst transfer rate was significantly higher in the patients who achieved live births than in the ones who did not (63.6% *vs.* 11.8%, *p*=0.010).

**Table 3:** Comparison of live births and non-live births in the younger low group.

	Live birth (n =11 patients)	Non-live birth (n = 17 patients)	*p*
Age (y), mean ± SD	35.40 ± 1.76	35.4±1.46	N.S.
No. of oocytes retrieved, mean ± SD	2.00 ± 2.30	3.00 ± 1.63	N.S.
Frequency of oocyte retrievals per patient, mean ± SD	3.45 ± 1.18	2.05 ± 0.95	0.035
No. of fertilized oocytes, mean ± SD	2.00 ± 1.99	2.00 ± 1.54	N.S.
Blastocyst transfer rate	63.6 % (7/11)	11.8 % (2/17)	0.010
Fresh embryo transfer	66.7 % (2/3)	100 % (1/1)	N.S.
Thawed embryo transfer	62.5 % (5/8)	6.3 % (1/16)	0.04

AMH=anti-Mullerian hormone; N.S.= not significant.

We further examined the blastocyst transfer rate by fresh and thawed embryo transfer cycles in a subset analysis. Table 3 shows the blastocyst transfer rates in the live birth and non-live birth groups further divided into fresh and thawed embryo transfer recipients in the younger low AMH group. In the fresh embryo transfer group, the blastocyst transfer rate was not significantly different between individuals with and without live births. However, in the thawed embryo transfer group, the blastocyst transfer rate was significantly higher in the live birth group than in the non-live birth group (*p*=0.04).

## DISCUSSION

To date, there has been no consensus on the relationship between AMH and pregnancy and live birth rates with ART, with some studies reporting correlations (Eldar-Geva *et al*., 2005; Nelson *et al*., 2007; Silberstein *et al*., 2006) and others reporting the lack thereof (Fanchin *et al*., 2007; Peñarrubia *et al*., 2005; Fiçicioglu *et al*., 2006; Smeenk *et al*., 2007). A recent meta-analysis examined the association between AMH and IVF treatment cycles with pregnancy and implantation rates, with results demonstrating only a weak association (Tal *et al*., 2015). Conversely, in the literature focusing on live births, a weak but relevant relationship (Tal *et al*., 2018) was reported; however, no consensus has been achieved.

Our findings indicate that AMH was not associated with live births in the younger group. However, the rate of live births and pregnancies was significantly higher in the older normal AMH group (AMH ≥1.0ng/mL). There have been reports associating AMH with live births and pregnancy rates in patients aged 38 years or older (Kato *et al*., 2015), which is consistent with the results of this study. The total HMG dose was significantly higher in the normal AMH group than in the low AMH group. Furthermore, the total HMG dose in the low AMH group was relatively lower owing to the implementation of a mild-stimulation protocol. Likewise, estrogen levels were lower in the low AMH group. Patients categorized into the younger low AMH group were few, and the number of patients in this study was also small, such that a multivariate analysis was not feasible. Therefore, a univariate analysis was instead conducted to compare patients who achieved live births with patients who did not. The rate of blastocyst transfer and the mean frequency of oocyte retrievals per patient were significantly associated with the characteristics of patients with live births in the younger low AMH group. Sefrioui *et al*. (2019) retrospectively examined IVF and pregnancy outcomes of 390 patients in the low AMH group (<0.4ng/mL) and 352 patients in the normal AMH group (1.3-2.6ng/mL). In patients aged ≤35years, the clinical pregnancy rates in the low AMH group and the normal AMH group were 27% and 41%, respectively. This indicates that younger women with low AMH have a good chance of getting pregnant (Sefrioui *et al*., 2019). In this study, the younger low AMH group had similar pregnancy rates and live birth rates compared with the younger normal AMH group.

Our report suggests that young people with low AMH have the potential to become pregnant if they can obtain blastocysts with frequent oocyte retrievals. Examination of the blastocyst transfer rates in fresh and thawed embryo transfers in the live birth and non-live birth groups in a subset analysis revealed significantly higher blastocyst transfer rates in the live birth group.

This study has some limitations. First, this study is retrospective in nature. Second, the type of ART treatment included both fresh embryo and thawed embryo transfers, which may have introduced bias based on the type of treatment. Further studies with large sample sizes are warranted to determine this clinically important issue and to strengthen the findings of our study.

## CONCLUSION

The live birth rate of younger patients with low AMH levels was equivalent to that of younger patients with normal AMH values. In addition, the likelihood of achieving a live birth increased with the number of blastocyst transfers. Thus, aggressive treatment intervention and continuation are considered acceptable.
